# Clinical application of next generation sequencing for Mendelian disease diagnosis in the Iranian population

**DOI:** 10.1038/s41525-024-00393-0

**Published:** 2024-02-19

**Authors:** Ayda Abolhassani, Zohreh Fattahi, Maryam Beheshtian, Mahsa Fadaee, Raheleh Vazehan, Fatemeh Ahangari, Shima Dehdahsi, Mehrshid Faraji Zonooz, Elham Parsimehr, Zahra Kalhor, Fatemeh Peymani, Maryam Mozaffarpour Nouri, Mojgan Babanejad, Khadijeh Noudehi, Fatemeh Fatehi, Shima Zamanian Najafabadi, Fariba Afroozan, Hilda Yazdan, Bita Bozorgmehr, Azita Azarkeivan, Shokouh Sadat Mahdavi, Pooneh Nikuei, Farzad Fatehi, Payman Jamali, Mahmoud Reza Ashrafi, Parvaneh Karimzadeh, Haleh Habibi, Kimia Kahrizi, Shahriar Nafissi, Ariana Kariminejad, Hossein Najmabadi

**Affiliations:** 1 Kariminejad - Najmabadi Pathology & Genetics Center, Tehran, Iran; 2 Genetics Research Center, University of Social Welfare and Rehabilitation Sciences, Tehran, Iran; 3 Genetic Clinic of Tehran Welfare Organization, Tehran, Iran; 4 Molecular Medicine Research Center, Hormozgan Health Institute, Hormozgan University of Medical Sciences, Bandar Abbas, Iran; 5 Nasle Salem Genetic Counseling Center, Bandar Abbas, Iran; 6 Department of Neurology, Neuromuscular Research Center, Shariati Hospital, Tehran University of Medical Sciences, Tehran, Iran; 7 Genetic Counseling Center, Shahroud Welfare Organization, Semnan, Iran; 8 Children’s Medical Center, Tehran University of Medical Sciences, Tehran, Iran; 9 Pediatric Neurology Department, Shahid Beheshti University of Medical Sciences, Tehran, Iran; 10 Hamedan University of Medical Science, Hamedan, Iran

**Keywords:** Genetic testing, Genetic testing, Genetics research

## Abstract

Next-generation sequencing (NGS) has been proven to be one of the most powerful diagnostic tools for rare Mendelian disorders. Several studies on the clinical application of NGS in unselected cohorts of Middle Eastern patients have reported a high diagnostic yield of up to 48%, correlated with a high level of consanguinity in these populations. We evaluated the diagnostic utility of NGS-based testing across different clinical indications in 1436 patients from Iran, representing the first study of its kind in this highly consanguineous population. A total of 1075 exome sequencing and 361 targeted gene panel sequencing were performed over 8 years at a single clinical genetics laboratory, with the majority of cases tested as proband-only (91.6%). The overall diagnostic rate was 46.7%, ranging from 24% in patients with an abnormality of prenatal development to over 67% in patients with an abnormality of the skin. We identified 660 pathogenic or likely pathogenic variants, including 241 novel variants, associated with over 342 known genetic conditions. The highly consanguineous nature of this cohort led to the diagnosis of autosomal recessive disorders in the majority of patients (79.1%) and allowed us to determine the shared carrier status of couples for suspected recessive phenotypes in their deceased child(ren) when direct testing was not possible. We also highlight the observations of recessive inheritance of genes previously associated only with dominant disorders and provide an expanded genotype–phenotype spectrum for multiple less-characterized genes. We present the largest mutational spectrum of known Mendelian disease, including possible founder variants, throughout the Iranian population, which can serve as a unique resource for clinical genomic studies locally and beyond.

## Introduction

Single-gene Mendelian disorders affect millions of individuals worldwide and account for an important public health burden as predominantly life-threatening or chronically debilitating conditions. Genetic diagnosis can benefit patients and their families by leading to appropriate therapy and providing insights into the prognosis and recurrence risk of the disorder within the family. However, establishing a definitive genetic diagnosis in many cases is a complicated and extended process, and a significant number of patients with presumed genetic disorders remain undiagnosed after receiving conventional clinical evaluation and genetic testing^1,2^.

In recent years, next-generation sequencing (NGS) has been proven to be one of the most powerful diagnostic tools for rare Mendelian disorders, especially genetically heterogeneous conditions. There are currently two general approaches in the clinical application of NGS assays. When a specific phenotype associated with a number of genes is suspected, targeted gene panel sequencing is applied, whereas exome sequencing (ES) is commonly implemented in the diagnostic evaluation of patients with a wide range of differential diagnoses or uncharacterized genetic diseases.

While the current diagnostic rate of NGS-based testing in unselected cohorts of patients generally ranges from 24% to 34%^3–9^, several studies in Middle Eastern populations have reported a higher yield of up to 48%^10–13^. The relatively higher yield in Middle Eastern patients is correlated with a high level of parental consanguinity and the predominance of autosomal recessive (AR) diagnoses in these populations. This signifies the implementation of NGS testing as a primary test of choice in countries within the so-called “consanguinity belt,” including Iran, with a 40% rate of consanguineous marriages^14,15^.

Despite the anticipated high frequency of AR disorders in the Iranian population, the focus has been mainly on common monogenic disorders such as thalassemia, cystic fibrosis, phenylketonuria, spinal muscular atrophy, duchene muscular dystrophy, and fragile X syndrome, which are detectable through conventional genetic testing. However, comprehensive data on the prevalence and genetic diversity of rare Mendelian disorders in this region is currently lacking, primarily due to limited access to advanced genome sequencing methods^16^. Consistent with increased affordability and access to NGS technologies in developing countries in recent years, NGS testing is becoming widely ordered by clinicians across Iran. This represented a unique opportunity to evaluate the diagnostic utility of this approach across different clinical indications in Iranian patients and allowed us to create the most comprehensive view of the mutational spectrum of known Mendelian disease throughout the country. We report a retrospective analysis of data from 1436 consecutive cases referred to our clinical diagnostic laboratory for ES or targeted gene panel testing over an 8-year period.

## Results

### Patient demographics

A total of 1436 Iranian index cases were referred for diagnostic NGS testing, with 1075 (74.9%) submitted for exome sequencing and 361 (25.1%) for targeted gene panel sequencing. NGS was performed on 1315 probands only (solo, 91.6%), 42 probands plus parents (trio, 2.9%), and 79 one or both healthy parents of the deceased affected child(ren) (parents, 5.5%). The majority of patient referrals (62.3%), encompassing 76.4% of ES cases (821/1075), were between 2018 and 2020. The breakdown of clinical indications in ES cases over time is presented in Supplementary Fig. 1.

The patient population comprised 777 males (54.1%) and 614 females (42.8%), in addition to 45 (3.1%) fetal cases from terminated pregnancies. The age of probands at testing, categorized into three age groups, ranged between prenatal to 72 years. Notably, parental consanguinity was present in the majority of cases (72.4%), while most patients (65%) had a negative family history (Table 1). Overall, 56% (805/1436) of patients presented with neurological conditions (Supplementary Fig. 2).

**Table 1:** Patient demographics and testing indications

Features	No. of cases (*N* = 1436)	% of total cases	No. of cases with positive results (*N* = 670)	% of positive results	Diagnostic rate in each category (%)
*Gender*					
Fetus	45	(3.1)	13	(1.9)	(28.9)
Female	614	(42.8)	283	(42.3)	(46.1)
Male	777	(54.1)	374	(55.8)	(48.1)
*Age at testing*					
Prenatal	45	(3.1)	13	(1.9)	(28.9)
Children (<5 yr)	374	(26.0)	164	(24.5)	(43.9)
Children and adolescents (5–18 yr)	492	(34.3)	248	(37.0)	(50.4)
Adults (>18 yr)	525	(36.6)	245	(36.6)	(46.7)
*Consanguinity*					
Yes	1040	(72.4)	505	(75.4)	(48.6)
No	396	(27.6)	165	(24.6)	(41.7)
*Family history*					
Positive	502	(35.0)	269	(40.1)	(53.6)
Negative	934	(65.0)	401	(59.9)	(42.9)
*Previous genetic testing*					
Yes	313	(21.8)	146	(21.8)	(46.6)
No	1123	(78.2)	524	(78.2)	(46.6)
*Referral date*					
2012^a^–2014	25	(1.8)	16	(2.4)	(64.0)
2015–2017	516	(35.9)	266	(39.7)	(51.5)
2018–2020^a^	895	(62.3)	388	(57.9)	(43.3)
*NGS test type*					
ES	1075	(74.9)	474	(70.8)	(44.1)
Targeted	361	(25.1)	196	(29.2)	(54.3)
*NGS test design*					
Solo	1315	(91.6)	618	(92.3)	(47)
Trio	42	(2.9)	13	(1.9)	(31.0)
Parents	79	(5.5)	39	(5.8)	(49.4)
*Clinical indication (top-level HPO term)*					
Abnormality of the nervous system	424	(29.5)	163	(24.3)	(38.4)
Abnormality of the musculature	387	(26.9)	211	(31.4)	(54.5)
Abnormality of the ear	116	(8.1)	58	(8.8)	(50.0)
Abnormality of metabolism/homeostasis	92	(6.4)	42	(6.3)	(45.6)
Abnormal central motor function	89	(6.2)	41	(6.1)	(46.1)
Peripheral neuropathy	87	(6.1)	37	(5.5)	(42.5)
Abnormality of the skin	71	(4.9)	48	(7.2)	(67.6)
Abnormality of the eye	55	(3.8)	23	(3.4)	(41.8)
Abnormality of the skeletal system	37	(2.6)	19	(2.8)	(51.4)
Abnormality of prenatal development or birth	33	(2.3)	8	(1.2)	(24.2)
Abnormality of blood and blood-forming tissues	17	(1.2)	11	(1.6)	(64.7)
Abnormality of the genitourinary system	14	(1.0)	5	(0.8)	(35.7)
Abnormality of connective tissue	10	(0.7)	3	(0.5)	(30.0)
Abnormality of the cardiovascular system	4	(0.3)	1	(0.1)	(25.0)

^a^Patient referral dates are from July 2012 to July 2020.

The distribution of patients’ major clinical indications based on HPO is presented in Table 1. The two most common testing indications, collectively accounting for more than half of all referrals, were abnormality of the nervous system (29.5%) and abnormality of the musculature (26.9%). Developmental delay and/or intellectual disability and seizures were the most frequent indications in patients grouped as an abnormality of the nervous system. Patients within the abnormality of the musculature group mainly presented with muscular dystrophy, myopathy, and myasthenic syndrome. The next most frequent indications for referral were abnormalities of the ear (8.1%), metabolism/homeostasis (6.4%), central motor function (6.2%), peripheral neuropathy (6.1%), skin (4.9%), and eye (3.8%). A complete list of the patient’s phenotypic and genotypic information is provided in Supplementary Table 2.

### Spectrum of identified variants

**Fig. 1:**
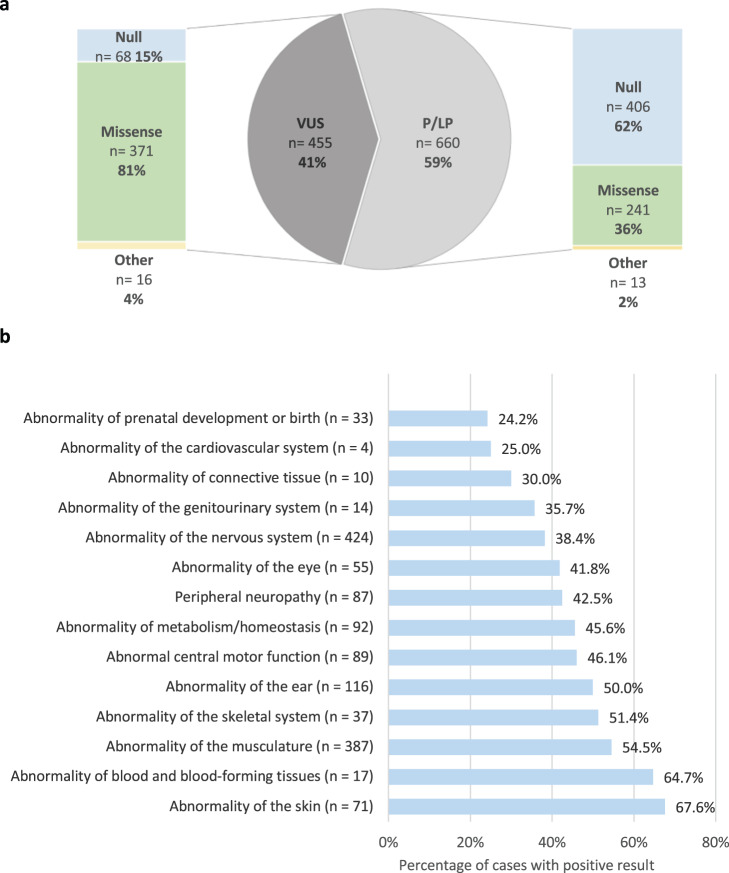
Characteristics of the reported variants and test yield based on patients’ phenotypes. **a** Distribution of the identified variants classified according to the ACMG guidelines and variant types. VUS variants of uncertain significance, P/LP pathogenic and likely pathogenic variants, Null nonsense, frameshift, ±1 or 2 splice sites, and initiation codon variants; Other, in-frame insertions or deletions, stop-loss, synonymous and promoter region variants. **b** Diagnostic yield based on major clinical indications (top-level HPO term).

A total of 1115 unique variants in phenotype-related genes were reported across 1005 cases with non-negative results, including 286 (25.7%) pathogenic variants, 374 (33.5%) likely pathogenic variants, and 455 (40.8%) variants of uncertain significance, classified according to the ACMG/AMP guidelines (Supplementary Table 2). Remarkably, about 55.6% (620/1115) of all the reported variants, including 241 (36.5%, 241/660) of the pathogenic and likely pathogenic variants, were novel as defined by not being previously reported in patients described in the literature. A broad spectrum of variant types was observed among all the reported variants, including 612 missense, 186 nonsense, 178 frameshift, 102 splice-site, 24 in-frame insertion or deletions, 8 initiation codon, 2 stop-loss, 2 promoter region, and 1 synonymous. Of 660P/LP variants, 406 (61.5%) were null variants (nonsense, frameshift, ±1 or 2 splice sites, initiation codon), 241 (36.5%) were missense variants, and 13 (2.0%) were other variant types (in-frame insertion or deletions, stop-loss, promoter region, synonymous) (Fig. 1a).

### Diagnostic yield

Overall, across the 1436 cases, a positive molecular diagnosis was reported in 46.7% (*n* = 670), a VUS result was reported in 22.1% (*n* = 318), an unclear result was reported in 1.2% (*n* = 17), and 30% (*n* = 431) received a negative result. There was a higher diagnostic yield from patients referred for targeted testing (54.3%, 196/361) compared to ES patients (44.1%, 474/1075). The frequency of positive results was the highest among cases performed as parents (49.4%, 39/79), followed by solo (47%, 618/1315) and trio cases (31%; 13/42) (Table 1). In the analysis of the diagnostic outcome in phenotype groups, the yield was highest for abnormalities of the skin (67.6%), blood and blood-forming tissues (64.7%), the musculature (54.5%), the skeletal system (51.4%), the ear (50%), central motor function (46.1%), and metabolism/homeostasis (45.6%). In contrast, abnormalities of the cardiovascular system (25%) and prenatal development/birth (24.2%) had the lowest diagnostic yield (Fig. 1b).

The distribution of the presumed mode of inheritance in positive cases is presented in Table 2. Autosomal recessive inheritance accounted for the majority of positive cases (79.1%, 530/670), followed by autosomal dominant inheritance (14.6%, 98/670) and X-linked inheritance (6.3%; 42/670). Of the 530 autosomal recessive diagnosed conditions, 441 (83.2%) were linked to homozygous variants, 50 (9.4%) demonstrated compound heterozygosity of two distinct variants, and 39 (7.4%) were cases of heterozygous variants identified in the parent(s) of a deceased patient. Among the 98 autosomal dominant diagnosed conditions, 46 (46.9%) were linked to a de novo variant, 9 (9.2%) were inherited from a symptomatic parent, 4 (4.1%) resulted from parental gonadal mosaicism, and 39 (39.8%) remained undetermined due to unavailability of parental samples. Of the 42 X-linked diagnosed disorders, 36 (85.7%) occurred in males and 6 (14.3%) in females; 15 X-linked alleles were inherited from healthy mothers, 7 resulted from de novo variants, 3 were inherited from a symptomatic parent, and 17 remained with unknown parental origin.

**Table 2:** Inheritance patterns among 670 positive cases

Mode of inheritance	No. of cases (%) *N* = 670
*Autosomal recessive*	530 (79.1%)
Homozygous	184
Apparently homozygous^a^	257
Compound heterozygous	27
Potential compound heterozygous^a^	23
Heterozygous in parent(s) of a deceased patient	39
*Autosomal dominant*	98 (14.6%)
De novo	46
Inherited from an affected parent	9
Parental gonadal mosaicism	4
Not studied^a^	39
X-linked	42 (6.3%)
X-linked recessive	32
De novo	3
Maternally inherited	15
Not studied^a^	14
X-linked dominant	10
De novo	5
Inherited from affected mother	3
Not studied^a^	2

^a^Parental inheritance was not determined due to the unavailability of samples.

^b^Includes two patients with both AD and AR disorders. cIncludes one patient with both XLD and AR disorders.

### Dual diagnoses

Among the 474 cases with a positive ES result, 11 patients (2.3%) received a dual molecular genetic diagnosis (targeted cases were excluded considering the limitations of panel sequencing to detect dual diagnosis comprehensively). The clinical and genetic information of these patients is provided in detail in Table 3 and Supplementary Table 3. The majority of patients in this group were considered to have overlapping phenotypes based on the observation of one or more clinical features associated with both molecular diagnoses, while patients for whom no phenotypic features were shared between molecular diagnoses were categorized as having two distinct phenotypes. Consanguinity was reported for the majority of these cases (81.8%, 9/11), and autosomal recessive phenotypes were expectably the most frequent observation in this group.

**Table 3:** Patients with dual molecular diagnosis

ID^a^	Consanguinty	Gene	Associated disease (OMIM ID)	Inheritance	Category
Patient 1	No	*SRCAP*	Floating-Harbor syndrome (136140)	AD	Overlapping
		*PTPN11*	Noonan syndrome 1 (163950)	AD	
Patient 2	Yes	*PNPLA8*	Mitochondrial myopathy with lactic acidosis (251950)	AR	Overlapping
		*SGCD*	Muscular dystrophy, limb-girdle, autosomal recessive 6 (601287)	AR	
Patient 3	Yes	*SLC22A5*	Carnitine deficiency, systemic primary (212140)	AR	Overlapping
		*DNMT3A*	Tatton–Brown–Rahman syndrome (615879)	AD	
Patient 4	Yes	*MYO7A*	Deafness, autosomal recessive 2 (600060)/ Usher syndrome, type 1B (276900)	AR	Distinct
		*BBS1*	Bardet-Biedl syndrome 1 (209900)	AR	
Patient 5	Yes	*SACS*	Spastic ataxia, Charlevoix-Saguenay type (270550)	AR	Distinct
		*HPS6*	Hermansky–Pudlak syndrome 6 (614075)	AR	
Patient 6	Yes	*NALCN*	Hypotonia, infantile, with psychomotor retardation and characteristic facies 1 (615419)	AR	Distinct
		*GALT*	Galactosemia (230400)	AR	
Patient 7	Yes	*ITGA7*	Muscular dystrophy, congenital, due to ITGA7 deficiency (613204)	AR	Overlapping
		*ATAD3A*	Harel-Yoon syndrome (617183)	AR	
Patient 8	Yes	*FKRP*	Muscular dystrophy-dystroglycanopathy (congenital with brain and eye anomalies), type A, 5 (613153)	AR	Overlapping
		*GYG1*	Polyglucosan body myopathy 2 (616199)	AR	
Patient 9	Yes	*HECW2*	Neurodevelopmental disorder with hypotonia, seizures, and absent language (617268)	AD	Overlapping
		*ERCC8*	Cockayne syndrome, type A (216400)	AR	
Patient 10	No	*CDKL5*	Developmental and epileptic encephalopathy 2 (300672)	XLD	Overlapping
		*POLG*	Mitochondrial DNA depletion syndrome 4A (Alpers type) (203700)	AR	
Patient 11	Yes	*PTPN11*	Noonan syndrome 1 (163950)	AD	Distinct
		*CAPN5*	Vitreoretinopathy, neovascular inflammatory (193235)	AD	

^a^Additional information about these cases appears in Supplementary Table 3.

### Important medical implications of diagnostic NGS

Medically actionable secondary findings in the ACMG-recommended list of 59 genes were analysed in 270 cases referred for ES testing. Reportable findings were identified in nine cases (3.3%), which include P/LP variants in *MYBPC3 (2), DSG2 (2), MUTYH, OTC, TNNI3, BRCA1 and BRCA2* (Supplementary Table 4). The positive diagnostic results had the potential to impact the treatment and clinical management of several patients. Selected examples included children diagnosed with Wilson disease (MIM:277900), DOPA-responsive Dystonia (MIM: 128230), Pyruvate dehydrogenase E1-alpha deficiency (MIM:312170), Pyridoxine-dependent Epilepsy (MIM: 266100), and Cerebral creatine deficiency syndrome 2 (MIM:612736). Furthermore, predictive testing for subsequent pregnancies (prenatal or preimplantation genetic diagnosis) was performed in 16% (*n* = 108) of families with positive diagnoses.

### Recurrent molecular findings

The majority of the positive cases (68.2%, 457/670) had P/LP variants in a gene at least twice observed in this series (117 genes). These genes were predominantly associated with neuromuscular disorders, sensorineural hearing loss, skin disorders and various neurodevelopmental phenotypes, providing insight into the prevalence of these genetic conditions among Iranian patients. The recurrently mutated genes in general and in each phenotypic group are listed in Supplementary Table 5 and Fig. 3. The most commonly diagnosed disorders in our cohort, each accounting for 1–5% of positive results, were muscular dystrophies related to *CAPN3* (*n* = 34), *DYSF* (*n* = 15), *LAMA2* (*n* = 12), *SGCA* (*n* = 11), and *DMD* (*n* = 22), *NF1*-related neurofibromatosis (*n* = 12), *ATM*-related ataxia (*n* = 11), *MYO7A*-related hearing loss (*n* = 10), *CHRNE*-related myasthenic syndrome (*n* = 9), *RYR1*-related congenital myopathy (*n* = 8), *SPG11*-related spastic paraplegia (*n* = 8), and *TYR*-related Albinism (*n* = 7).

Furthermore, of the reported recessive P/LP variants, 59 were identified in two or more unrelated positive cases, and 35 were present in the heterozygous state in at least one healthy Iranian individual in the Iranome database^17^ (Supplementary Table 6). These variants, collectively accounting for 14.2% (94/660) of all the identified P/LP variants, suggest presumptive founder effects in the Iranian population that require additional haplotype and population-specific analysis for confirmation. Notably, among these presumed founder variants, 28 were absent from other population databases, including gnomAD and the Greater Middle East (GME) variome^18^. This subset encompasses 19 novel or rare recessive pathogenic variants exclusively reported in Iranian patients up to the present, which could be denoted as “Iranian-only” mutations. It is noteworthy that several of these variants were novel missense variants that could be classified as likely pathogenic only through observation in two unrelated cases with the same phenotypic presentation. These variants include *MME* c.499T>A (p.Trp167Arg), *SGCA* c.113A>G (p.His38Arg), *FKRP* c.1034G>C (p.Gly345Ala), *ASAH1* c.109C>A (p.Pro37Thr)^19^, and *OTOA* c.1727T>C (p.Ile576Thr).

### Unexpected inheritance patterns and molecular events

The predominance of consanguineous families in this cohort led to the identification of bi-allelic variants in 11 genes with only an autosomal dominant pattern of inheritance in the OMIM database at the time of reporting. These genes were either not previously associated with any AR disorders or were reported with recessive inheritance only in extremely rare cases in the literature (Table 4). The clinical presentations in the majority of cases in this series were either similar to or more severe than the established dominant disorder. For example, in three separate cases with variants in *TBX4*, *GLI3* (both published elsewhere^20,21^), and *BICC1* genes, a severe embryonic condition was observed in the homozygous fetuses, while the heterozygous parents were either mildly affected or unaffected. Other remarkable examples include recessive forms of *DCTN1*-related neurodegenerative disorder, *KCNC3*-related spinocerebellar ataxia, *HARS1*-related Charcot-Marie-Tooth disease^22^, and *MITF*-related hearing loss^23^. Unlike previous observations of biallelic *PKD1* variants in severe pediatric Polycystic Kidney Disease^24,25^, a homozygous missense variant in this gene was identified in a 33-year-old patient presenting with bilateral cystic kidneys, hypertension, urinary issues, and an apparently negative family history. This finding is consistent with the published literature on biallelic hypomorphic *PKD1* alleles leading to a mild adult-onset phenotype^26^. In addition, a distinct and only recently reported recessive phenotype for the *UFSP2* gene was observed in a patient presenting with global developmental delay, intractable epilepsy, and brain atrophy^27^. Variants in this gene were previously associated with autosomal dominant skeletal system disorders without neurological dysfunction.

**Table 4:** Recessive inheritance in dominant disease genes

Patient no.	Observed recessive phenotype	Gene	Zygosity	Variant(s)	Dominant phenotype (OMIM)	Parental health status	Comparison with AD phenotype	Previous reports of recessive inheritance
574	Absent lower limbs; Absent pelvic bones; Hypoplastic sacrum; Severe lung hypoplasia; Ambiguous genitalia	*TBX4*	Hom	NM_001321120.2c.339T>A p.(Tyr113*)	Ischiocoxopodopatellar syndrome with or without pulmonary arterial hypertension	Mildly affected	More severe	No^a^
751	Multiple skeletal abnormalities of lower limbs with bowing of femurs and polydactyly and syndactyly of all four limbs; Imperforate anus; Rectal stenosis; Anocutaneous fistula; Polysplenia	*GLI3*	Hom	NM_000168.5c.1927C>T p.(Arg643*)	Greig cephalopolysyndactyly syndrome Pallister–Hall syndrome Polydactyly, postaxial, types A1 and B Polydactyly, preaxial, type IV	Mildly affected	More severe	No^a^
1427	Aborted fetus with polycystic kidney	*BICC1*	Hom	NM_001080512.3c.611C>T p.(Pro204Leu)	Renal dysplasia, cystic, susceptibility to	Healthy	More severe	No
647	Onset at the age of 21-year with a speech problem and attention deficit; Mild muscle spasticity, upper limbs, mild; Drooling; Parkinsonian gait; Aggression; Brain MRI: generalized atrophic changes	*DCTN1*	Hom	NM_004082.5c.2267C>T p.(Ala756Val)	Neuronopathy, distal hereditary motor, type VIIB Perry syndrome Amyotrophic lateral sclerosis, susceptibility to	Unknown^b^	Similar	No
1091	Developmental regression; Hearing loss, severe to profound; Abnormal teeth; Asymmetric kidneys; Loss of ambulation	*KCNC3*	Hom	NM_004977.3c.2060C>T p.(Pro687Leu)	Spinocerebellar ataxia 13	Unknown^b^	Similar	No
41	Recurrent bone fractures; Growth failure; Delayed puberty	*COL1A2*	Hom	NM_000089.4c.910G>A p.(Gly304Ser)	Combined osteogenesis imperfecta and Ehlers–Danlos syndrome 2 Ehlers–Danlos syndrome arthrochalasia type, 2 Osteogenesis imperfecta, type II Osteogenesis imperfecta, type III Osteogenesis imperfecta, type IV	Mildly affected	More severe	Yes
1077	Face and hands Lesions; Skin biopsy: more compatible with progeria	*PPOX*	Hom	NM_000309.5c.848T>C p.(Ile283Thr)	Porphyria variegata	Unknown^b^	More severe	Yes
1172	Hypertension; Cystic kidney, bilateral; Urinary tract infections	*PKD1*	Hom	NM_001009944.2c.1360C>T p.(Arg454Cys)	Polycystic kidney disease 1	Unknown^b^	Similar	Yes
1068	Lower & upper limbs neuropathy; Difficulty walking; EDX: severe chronic demyelinating sensory-motor polyneuropathy with secondary axonal loss	*HARS1*	Compound Het Compound Het	NM_002109.6c.90+4A>C NM_002109.6c.1393A>C p.(Ile465Leu)	Charcot-Marie-Tooth disease, axonal, type 2W	Unknown^b^	Similar	Yes
1372	Hearing loss	*MITF*	Hom	NM_000248.4c.721T>C p.(Trp241Arg)	Tietz albinism-deafness syndrome Waardenburg syndrome, type 2A	Unknown^b^	Milder (Non-syndromic)	Yes
1430	Onset at the age of 4 months with Intractable epilepsy, infantile spasm; Developmental delay; Mental retardation; Impaired swallowing (NGT feeding); Brain MRI showed brain atrophy suggestive of neurodegenerative disorders; Abnormal EEG	*UFSP2*	Hom	NM_018359.5c.344T>A p.(Val115Glu)	Hip dysplasia, Beukes type Spondyloepimetaphyseal dysplasia, Di Rocco type	Unknown^b^	Distinct	Yes

*Hom*, homozygous, *Compound het*, compound heterozygous.

^a^These cases have been previously published elsewhere^20,21^.

^b^These individuals were reportedly healthy but had not undergone post-test clinical evaluation.

Furthermore, despite the initial assumption of a recessive genetic condition in 3 families with multiple similarly affected children and healthy parents, we identified heterozygous pathogenic variants in AD disease genes (*SETBP1, CRYAA, SMCHD1*) in the affected children but neither of the parents, indicating possible parental gonadal mosaicism. (patients 184, 794, 1003 in Supplementary Table 2). Consanguineous marriage suggestive of a typical AR pedigree was interestingly reported in two of these families. Identification of de novo variants in AR disease genes was another unusual molecular event observed in 3 patients who were each compound heterozygote for two P/LP variants in *SGCA, ASPM*, and *ABCA4* genes. In these individuals, one of the variants was parentally inherited while the other had arisen de novo (patients 36, 253, 398 in Supplementary Table 2).

### Expanding the mutational and phenotypic spectrum of less characterized genes

Variants in genes with limited mutational/phenotypic evidence in the literature or with OMIM phenotypes based on a single study were identified in 11 patients (*MRPS34, FIBP, LRP4, ZBTB11, CRIPT, BCKDK, PLAA, CC2D1A, TUBB2A, SOD1*). A detailed description of molecular findings and observed phenotypes in these patients are provided in Supplementary Table 7. These include the third so far described patients with *FIBP*-related Thauvin–Robinet–Faivre syndrome and *LRP4*-related congenital myasthenic syndrome, the third variant so far associated with *SOD1*-related spastic tetraplegia and axial hypotonia, and the first truncating variant associated with *TUBB2A*-related cortical dysplasia with other brain malformations. Notably, a possible expanded phenotype was observed in a patient with a homozygous *MRPS34* variant presenting with chronic motor neuropathy, which deviates significantly from the delayed psychomotor development reported in a single study for this gene^28^. Similarly, a milder clinical presentation for *PLAA*-related disorder was observed in two siblings with developmental delay, seizures, and hypotonia.

## Discussion

Large-scale clinical genomic studies have significantly contributed to our understanding of the clinical relevance of human genetic variation. While studies in outbred populations predominantly contribute to the better diagnosis of autosomal dominant genetic diseases, Middle Eastern populations with high rates of consanguinity have been proven as a valuable resource for the genetic analysis of recessive disorders. Despite the abundance of published research studies using NGS in Iranian patients, especially for novel disease gene discovery^29,30^, the clinical utilization of this technology on an unselected group of patients and the overall distribution of known Mendelian disorders in this ethnically diverse population is lacking.

We present the results of diagnostic NGS-based testing in 1436 consecutive cases from a clinically heterogenous cohort of patients in Iran. Establishing the molecular diagnosis of over 342 known disorders in 670 cases, we achieved an overall diagnosis rate of 46.7%. Furthermore, in 22.1% of cases, we identified clinically relevant VUS with strong evidence supporting pathogenicity but lacking sufficient data to be classified as P/LP based on ACMG/AMP criteria. Segregation analysis in the family and additional clinical and functional information could provide the evidence required to re-classify these variants either as P/LP or likely benign. Our overall diagnostic yield is similar to previously reported large heterogenous clinical series in consanguineous Middle Eastern patients^10–13^, while it is higher than that of studies in outbred populations^3–9^, further supporting the positive impact of consanguinity on diagnostic yield. Other possible contributing factors include the early use of NGS testing in the diagnostic process in the majority of cases, different categories of indications, differences in sample size, the population’s genetic background, or other properties of the cohort.

As expected, targeted sequencing of small gene panels, most commonly ordered for highly specific conditions such as epidermolysis bullosa, osteogenesis imperfecta, albinism, non-syndromic ichthyosis, etc., had a higher diagnosis rate than ES testing, which was typically ordered for more complex phenotypes and highly heterogenous groups of disorders. Furthermore, among patients with neurological conditions, those presenting with more specific neurological symptoms (e.g., ataxia, spastic paraplegia, sensory and motor and neuropathies) had the highest molecular diagnosis rate (46.9%, 139/296), likely due to facilitated genotype–phenotype correlation analysis^4^ (Supplementary Fig. 2). In line with previous reports^6,12^, we observed a statistically higher diagnostic yield in patients with positive family history (53.6% vs. 42.9%, *p* = 0.0002) and consanguinity (48.6% vs. 41.7%, *p* = 0.024). However, a lower diagnosis rate was achieved in our trio exome analysis compared to proband-only cases, which could be explained by the bias toward ordering trio testing for the nonconsanguineous families, and usually as a second-tier test.

Although the numbers are still modest for some clinical indications, a diagnostic yield of at least 24% is observed in all phenotypic groups. While disorders of the skin, blood, musculature, skeletal system, ear, metabolism, central motor function, peripheral nervous system, and eye have a high diagnostic yield (41–68%), other indications such as abnormality of prenatal development and the cardiovascular system show low diagnostic yield (<30%). The lack of sufficient phenotyping, as well as the higher likelihood of the presence of disease-causing structural genetic variations that remain undetectable by the NGS technique, might explain the lower yield obtained in patients in the latter two groups. The same trend and a low detection rate for isolated/less characterized phenotypes have been reported by previous studies^6,8^. Moreover, due to the shifts in referral patterns and the changing composition of high and low-yield phenotypes, we observed a decrease in the general diagnostic yield of ES over time. Specifically, referrals related to Musculature decreased from 79% to 20%, while cases associated with prenatal development increased from 0% to 4% of our patient cohort in the first to the last two years of the study period (Supplementary Fig. 1). This observation underscores that while the growing number of established disease genes over time can increase the diagnosis rate of ES in specific patient groups, a significant portion of diagnostic success within certain phenotypes relies on already-known genes.

The highly consanguineous nature of this cohort led to several significant outcomes. First, it resulted in the diagnosis of autosomal recessive disorders in the majority of patients (79.1%), with the enrichment for homozygous P/LP variants (83.2%). Interestingly, we identified rare homozygous P/LP variants in 37 ostensibly non-consanguineous cases, which could be attributed to either a founder effect or hidden consanguinity in populations such as Iran with a historical prevalence of intrafamily and interclan marriages, where familial relationships may be too distant to appear in the pedigree. The majority of seemingly unrelated parents within this group originated from the same rural region or town, suggesting regional clustering of private recessive pathogenic variants^30,31^. Moreover, we observed the recurrence of the identical recessive P/LP variants in multiple unrelated patients, indicating presumed founder variants in the Iranian population, which could potentially serve as a unique resource for population-based screening programs. Second, it facilitated determining the shared carrier status of the majority of couples (49.4%, 39/79) referred for the diagnosis of a suspected recessive phenotype observed in their deceased child(ren), when there was no access to their sample for direct testing. The practical and high-yield advantage of duo parental analysis in investigating the cause of fetal demise or premature death (referred to as molecular autopsy by proxy) has been previously reported in consanguineous populations^25,32^. Consistent with our results, the diagnostic rate in this group of cases has been reported to be higher compared to the general diagnostic yield in the same population, reaching 63% through reanalysis and novel gene discovery methodology^25^. Therefore, this approach holds promise in providing accurate genetic counseling and informed reproductive choices among comparable inbred families. Nevertheless, the lack of direct confirmation of the candidate variant in the deceased index remains a notable limitation of this method. Third, it led to the observation of several instances of homozygous variants in genes previously associated only with dominant disorders. These cases have the potential to advance our understanding of the mutational mechanisms (loss of function, gain of function, dominant negative) of associated phenotypes, which can further improve variant interpretation and genotype–phenotype correlation analysis^33^. Finally, we identified novel variants in ultra-rare recessive disorders, expanding the genetic and phenotypic spectrum of less-characterized genes and phenotypes. The value of ES in characterizing patients with multiple diagnoses and those with clinically actionable secondary findings has been previously emphasized^34,35^. In this study, we observed dual molecular diagnoses in 2.3% of positive ES cases and identified secondary findings in 3.3% of the analyzed cases, a frequency comparable to previously reported studies^10,36^.

While AR disorders accounted for the majority of diagnoses in this cohort, we observed autosomal dominant conditions in 14.6% of patients. An increase in this rate is expected upon parental testing and confirmation of the de novo occurrence of additional heterozygous variants reported as VUS in several patients. It is noteworthy that we unexpectedly identified dominant de novo causative variants in 17 cases with positive parental consanguinity, two of which had occurred in typical AR pedigrees due to possible germline mosaicism in either parent. In addition, among consanguineous cases with a dual molecular diagnosis, two patients were diagnosed with both dominant and recessive phenotypes, and in one patient two de novo causative variants in different dominant disease genes were identified. These observations highlight the importance of the application of all inheritance patterns when testing patients with a known family history of consanguinity.

Proband-only analysis performed in 91.6% of our cases demonstrated the high diagnostic utility of this strategy in a consanguineous population. While trio testing is the most commonly recommended strategy that allows the identification of de novo variants and phasing of recessive variants, its high cost remains a major financial constraint in countries like Iran, where there is no medical insurance coverage for genetic testing. The superiority of parent–child trios over solo analysis is mainly demonstrated in outbred populations where the majority of genetic diagnoses are attributed to de novo dominant or recessive compound heterozygous disease alleles. While in inbred populations with enrichment for homozygous recessive disorders, variant phasing at the time of interpretation is not considered an important determinant factor for better diagnosis. However, it is noteworthy that solo testing misses the potential preventive utility of parent–child trios in informing consanguineous parents about the 3% residual risk for additional autosomal recessive disorders in their offsprings^37^.

The comparable diagnostic yields of first-tier and reflex exome sequencing in our study (365/835 (43.7%) vs. 109/240 (45.4%), *p* = 0.67) highlight the cost-saving benefit of this testing strategy when applied as a first-line diagnostic approach. Moreover, while previous studies have suggested the application of broadly designed panels or sub-exome approach (medical exome) as cost-effective solutions in countries with limited resources^7,13^, performing ES and limiting the analysis to established disease genes offers an advantage by providing an opportunity for more resolved cases upon reanalysis of existing sequencing data through a combined diagnostic/research approach.

As a reference laboratory, we lacked access to post-test clinical follow-up data necessary to evaluate clinical management changes in patients with positive diagnoses. Additionally, the diagnostic yield in our center would have been affected by the low quality and quantity of available clinical information in test orders and the lack of communication between clinicians and interpreters. This information can facilitate clinicopathologic correlation analysis and variant interpretation by enabling comparisons with previously reported cases. The partnership of the clinician with the molecular laboratory has been previously shown to increase diagnostic yield^38^. Therefore, post-test diagnostic assessments, including biochemical and radiological tests, complementary molecular tests (e.g., deletion and duplication analysis by multiplex ligation-dependent probe amplification (MLPA)), and segregation analysis within the extended family, can help diagnose numerous additional cases. For instance, in two families with single P/LP variants identified by exome sequencing in AR genes, large multi-exon deletions on the second allele were detected by further MLPA analysis (patients 288 and 1335 in Supplementary Table S2). In addition, in two patients with heterozygous likely pathogenic variants in the *SMCHD1* gene, identified by ES, additional haplotype and DNA methylation analysis confirmed the digenic inheritance and thereby the diagnosis of Facioscapulohumeral muscular dystrophy 2 (FSHD2) (patients 184 and 744 in Supplementary Table 2).

Technical limitations may also account for a small but considerable fraction of our nondiagnostic cases. The presence of pathogenic copy number variation (CNV) may have been missed due to the non-optimized pipeline for CNV analysis. Considering the recent developments in algorithms for exon-level CNV detection by NGS, we anticipate an increase in the number of diagnosed patients upon reanalysis of the current cohort. This increase is especially expected for patients who received unclear diagnoses and who were carriers of P/LP variants in clinically relevant recessive genes, while a second variant was not detected. Moreover, considering the increasing number of newly discovered disease genes, improvements in bioinformatics algorithms, and new clinical manifestations in patients, we expect the annual reanalysis of ES data will continue to provide additional diagnoses. Nevertheless, other genetic changes such as translocations or inversions, repeat expansions, and alterations in intergenic, intronic, or regulatory regions remain undetected by exome or targeted sequencing. Our pipeline was also not designed or validated for detecting somatic mutations and mtDNA variants.

In conclusion, our data demonstrated the high diagnostic utility of NGS-based testing for a wide range of unselected phenotypes in Iranian patients. We identified recurrently diagnosed rare Mendelian disorders, shedding light on the prevalence of these genetic conditions in Iran. We show that proband-only genomic testing is an efficient and cost-effective diagnostic strategy in resource-limited countries with a high rate of consanguineous marriage and homozygous recessive genetic diseases. In addition to providing an expanded genotype–phenotype spectrum for less-characterized genes, we report observations of recessive inheritance of genes previously associated only with dominant disorders. Moreover, we highlight the potential of consanguineous populations for the diagnosis of suspected recessive conditions in deceased children by duo analysis of their parents. Our cohort’s genotypic and phenotypic data can serve as a unique resource for clinical genomic studies locally and beyond.

## Methods

### Patient population

This study includes 1546 sequenced samples comprising 1436 cases with suspected Mendelian disorders referred for clinical NGS testing at the Kariminejad-Najmabadi Pathology & Genetics Center, Tehran, Iran, from July 2012 to July 2020. Patients were referred by their clinicians either for ES or targeted gene panel sequencing. The study included 89 (6%) previously published cases.

For the majority of patients, proband-only testing was requested, whereas, for non-consanguineous families with negative family histories, clinicians were encouraged to consider trio-based sequencing. For couples (most commonly consanguineous) with suspected recessive phenotypes in their deceased children, NGS was performed on either or both individuals to determine their shared carrier status. In the earlier stages of the study, to minimize the financial burden on families, we exclusively performed targeted and exome sequencing on the mother, while the father’s analysis involved investigating the maternal candidate variant(s) through Sanger sequencing. This approach was later replaced by duo parental exome sequencing.

Samples were collected from index cases and available family members in the form of peripheral blood, tissue for fetal tissue samples obtained at autopsy, or extracted DNA samples. Pre- and post-test genetic counseling was provided by certified genetic counselors and/or a clinical geneticist. The clinical information provided by the referring clinicians, along with a family pedigree and test histories, e.g., MRI or genetic/metabolic, were carefully reviewed and collected.

We categorized patients’ clinical indications using top-level branching of Human Phenotype Ontology (HPO) nomenclature. In cases with multiple clinical features, the most medically impactful feature was considered for phenotypic categorization. Patients with developmental delay/intellectual disability often co-presented with other congenital anomalies, and multiple/specific neurological diagnoses such as seizures and autism spectrum disorder were categorized as “Abnormality of the nervous system.” Suspected motor neuron disease and movement disorders such as Spinal Muscular Atrophy (SMA), Amyotrophic lateral sclerosis (ALS), Hereditary Spastic Paraplegia (HSP), Ataxia, and Parkinson’s disease were grouped as “Abnormal central motor function.” For fetuses with multiple malformations, “Abnormality of prenatal development or birth” was selected. The referred patients either had previously undergone non-diagnostic genetic testing (karyotype, whole genome array CGH, molecular testing for specific genes) or were considered for NGS testing as a first-tier diagnostic approach.

### Ethics approval and consent to participate

This study was approved in Iran by the ethics committee of the Kariminejad-Najmabadi Pathology and Genetics Center. Written informed consent was obtained from all patients or their guardians. The study was carried out in accordance with the declaration of Helsinki.

### Exome/targeted sequencing and data analysis

Genomic DNA was extracted, and sequencing was performed by applying exome or targeted NGS protocols. For 1075 ES cases, the exome target regions were captured using Agilent SureSelect Human All Exome V4, V5, V6, and V7 Kits (Agilent Technologies, Inc., Santa Clara, CA, USA) or Twist Human Core Exome (Twist Bioscience, San Francisco, CA, USA). For 312 targeted cases, a custom-designed SureSelect hybrid capture panel targeting the coding regions of 685 confirmed disease-causing genes was used. For 49 targeted hearing loss cases, the OtoSCOPE® v6, v7, and v8 genetic testing platforms were used^39^. Paired-end sequencing was performed on Illumina sequencers (HiSeq 2000/2500/4000 and NovaSeq 6000) (Illumina, San Diego, CA, USA) according to the manufacturer’s protocol.

FASTQ files were mapped to the GRCh37/hg19 human reference sequence using Burroughs Wheeler Aligner (BWA)^40^. BAM processing, quality control, and coverage assessment were performed using Picard tools and the Genome Analysis ToolKit (GATK), adhering to the GATK best practices recommendations^41^. Variant calling was performed using GATK Haplotype Caller. An average coverage depth of 126X and 522X was obtained for exome and targeted sequencing samples, respectively, with 97.5% of the targeted regions (protein-coding exons based on CCDS and ±10 bp of flanking intronic sequence) sequenced at 10X and higher.

### Variant analysis, interpretation, and reporting

Variants were annotated and filtered using Annovar^42^ and an in-house bioinformatics tool. For visual verification of alignments, Integrative Genomics Viewer^43^ was used. Common variants (≥1% in the general population) and recurrent artifact variant calls were filtered out based on the latest available versions of 1000G (http://www.1000genomes.org), the Exome Variant Server (http://evs.gs.washington.edu), the Exome Aggregation Consortium database (EXAC) (http://exac.broadinstitute.org), the Genome Aggregation Database (gnomAD) (https://gnomad.broadinstitute.org), Iranome (http://www.iranome.ir) and internal databases.

All intronic variants located outside the boundaries of 10 bp from the exons and synonymous variants, except those located at exon/intron boundaries, were filtered out. Variants were then prioritized considering inheritance patterns, phenotype compatibility, population frequencies, variant types, and in silico prediction scores based on the information obtained from several resources, including the Online Mendelian Inheritance in Man (OMIM), ClinVar, HGMD, PubMed and using a number of in silico prediction tools (SIFT, Polyphen-2, MutationTaster, CADD, dbscSNV among others).

For targeted testing, only the variants within the requested gene panel, out of the 30 offered clinically themed gene panels, were analyzed (Supplementary Table 1). For ES analysis, variants in the internally developed phenotype-associated gene lists were considered initially, and if no candidate variant was identified, continued analysis for all known disease-causing genes in the OMIM database was performed. For determination of the shared carrier status of a couple for a suspected recessive phenotype in their deceased child (ren) within the duo parental sequencing group, only the variants identified within mutual genes between both parents were considered for analysis, with additional consideration given to X-linked recessive (XLR) genes in the female parent. In single-parent sequencing cases, our analysis was restricted to variants within clinically relevant AR and XLR genes identified in the female parent, and the candidate variant(s) were further investigated through Sanger Sequencing in the male parent.

The analysis of secondary findings began in September 2019, coinciding with a delay in the accreditation of clinical exome sequencing in Iranian laboratories. ES results were analyzed for known or expected pathogenic variants in 59 medically actionable genes in accordance with the latest recommendations of the ACMG (ACMG SF v2.0)^35^. Prior to the specified variants in these genes were exclusively analyzed when relevant to the patient’s phenotype.

The selected list of candidate variants in each patient was re-evaluated by a local clinician to determine those most relevant to the patient’s phenotype, and the final candidate variants were classified according to the American College of Medical Genetics and Genomics/Association for Molecular Pathology (ACMG/AMP) guidelines and the Clinical Genome (ClinGen) recommendations for using ACMG/AMP criteria^44,45^. The results were classified into the following four categories.

1. Positive: Pathogenic or likely pathogenic (P/LP) variant(s) in an established disease-causing gene associated with the reported clinical indication and present in the expected zygosity. Results including assumed compound heterozygous variants with at least one variant classified as P/LP, and those with pathogenic or likely pathogenic variant(s) in more than one gene (Dual diagnoses), are also included in this category.
2. VUS: Variant(s) of uncertain significance (VUS) in an established disease-causing gene associated/possibly associated with the reported clinical indication and present in the expected zygosity. This category includes VUS strongly suspected to be pathogenic but currently lacking sufficient evidence to be classified as P/LP by ACMG/AMP criteria.
3. Unclear: Single heterozygous P/LP variant in an established disease-causing gene with autosomal recessive phenotype compatible with the patient’s clinical indication.
4. Negative: No VUS/P/LP variants were identified in genes associated with the reported clinical indication.

### Variant confirmation and segregation analysis

All the candidate variants detected by NGS were confirmed by conventional PCR amplification and Sanger sequencing. Parents and available healthy/affected family members were also tested by Sanger sequencing for segregation analysis and determination of the origin and phase of variants. Short tandem repeat testing was additionally performed to confirm parentage for apparent de novo variants.

### Statistical analysis

The significance of the differences in diagnostic rate was analyzed with the Pearson chi-square test using SPSS. A *p*-value of 0.05 was used as a significance threshold.

### Reporting summary

Further information on research design is available in the Nature Research Reporting Summary linked to this article.

### Supplementary information

REPORTING SUMMARY

Supplementary Figures

Dataset 1: Supplementary Tables

## Data Availability

The datasets used and/or analysed during the current study are available from the corresponding author on reasonable request. All P/LP variants identified in this study have been submitted to the ClinVar database (Submission IDs: SUB10350875, SUB13967151), and accession numbers are provided in Supplementary Table 8.
