# Supplementary material for: Clinical application of next generation sequencing for Mendelian disease diagnosis in the Iranian population
Source: NPJ Genom Med. 2024 Feb 19;9:12. doi: 10.1038/s41525-024-00393-0 (PMC10876633; doi:10.1038/s41525-024-00393-0)
Supplement: Supplementary file 2 — Supplementary Figures [file 41525_2024_393_MOESM2_ESM.pdf]

Clinical application of next generation sequencing for Mendelian disease diagnosis in the Iranian population

Ayda Abolhassani[1], Zohreh Fattahi[1,2], Maryam Beheshtian[1], Mahsa Fadaee[1], Raheleh Vazehan[1], Fatemeh Ahangari[1], Shima Dehdahsi[1], Mehrshid Faraji Zonooz[1], Elham Parsimehr[1], Zahra Kalhor[1], Fatemeh Peymani[1], Maryam Mozaffarpour Nouri[1], Mojgan Babanejad[2], Khadijeh Noudehi[1], Fatemeh Fatehi[1], Shima Zamanian Najafabadi[1], Fariba Afroozan[1], Hilda Yazdan[1], Bita Bozorgmehr[1], Azita Azarkeivan[1], Shokouh Sadat Mahdavi[3], Pooneh Nikuei[4,5], Farzad Fatehi[6], Payman Jamali[7], Mahmoud Reza Ashrafi[8], Parvaneh Karimzadeh[9], Haleh Habibi[10], Kimia Kahrizi[2], Shahriar Nafissi[6], Ariana Kariminejad[1], Hossein Najmabadi[1,2,*]

[1]Kariminejad - Najmabadi Pathology & Genetics Center, Tehran, Iran. [2]Genetics Research Center, University of Social Welfare and Rehabilitation Sciences, Tehran, Iran. [3]Genetic Clinic of Tehran Welfare Organization, Tehran, Iran. [4]Molecular Medicine Research Center, Hormozgan Health Institute, Hormozgan University of Medical Sciences, Bandar Abbas, Iran. [5]Nasle Salem Genetic Counseling Center, Bandar Abbas, Iran. [6]Department of Neurology, Neuromuscular Research Center, Shariati Hospital, Tehran University of Medical Sciences, Tehran, Iran. [7]Genetic Counseling Center, Shahroud Welfare Organization, Semnan, Iran. [8]Children's Medical Center, Tehran University of Medical Sciences, Tehran, Iran. [9]Pediatric Neurology Department, Shahid Beheshti University of Medical Sciences, Tehran, Iran. [10]Hamedan University of Medical Science, Hamedan, Iran.

*Correspondence: Hossein Najmabadi; E-mail: hnajm12@yahoo.com

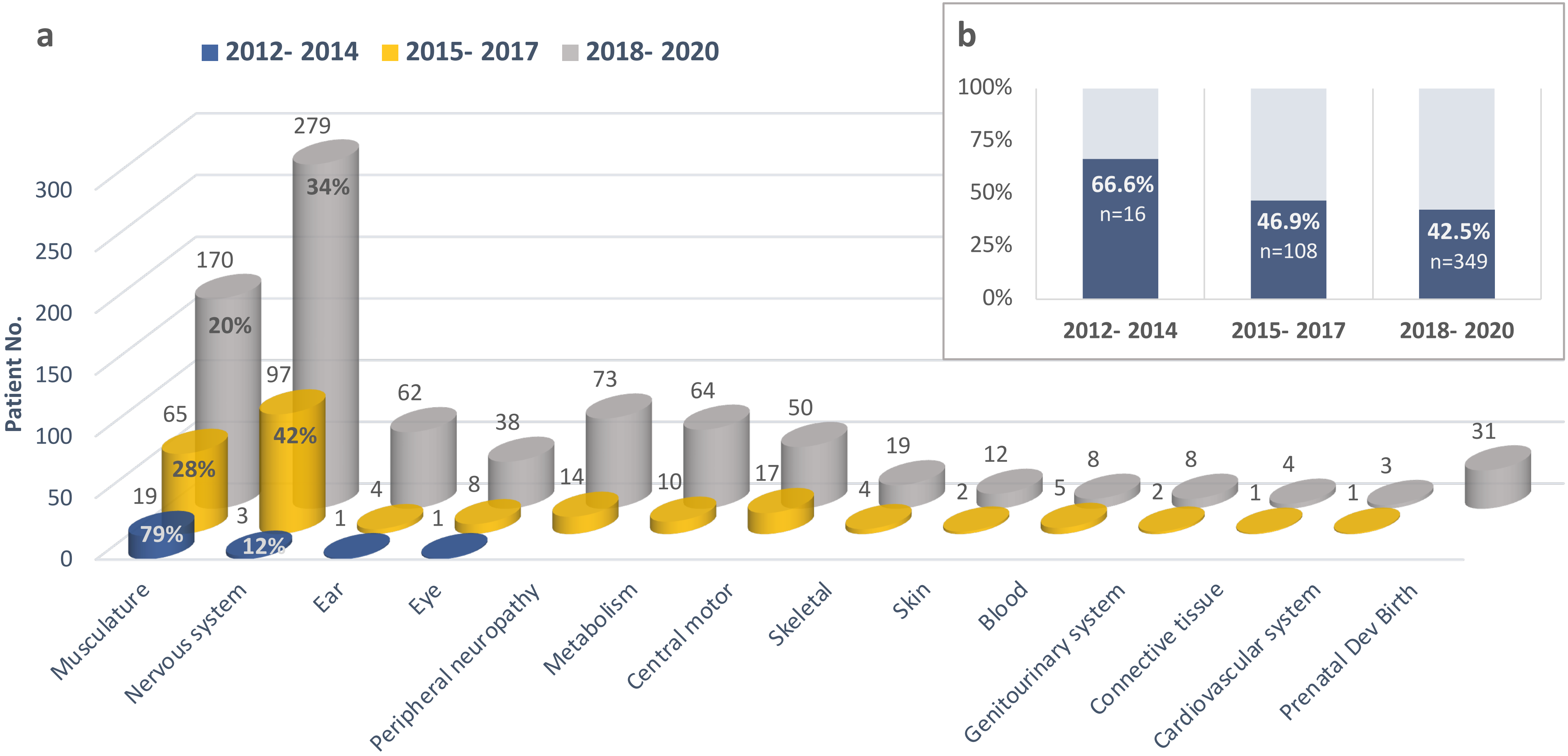

Supplementary Figure 1. The breakdown of clinical indications and diagnostic yield in exome sequencing (ES) referrals over time.
a Major clinical indications in 1075 patients referred for ES from July 2012 to July 2020 shown in three time intervals: (2012-2014; n = 24), (2015-2017; n = 230), and (2018-2020; n = 821). **b** Diagnostic yield of ES by referral dates.

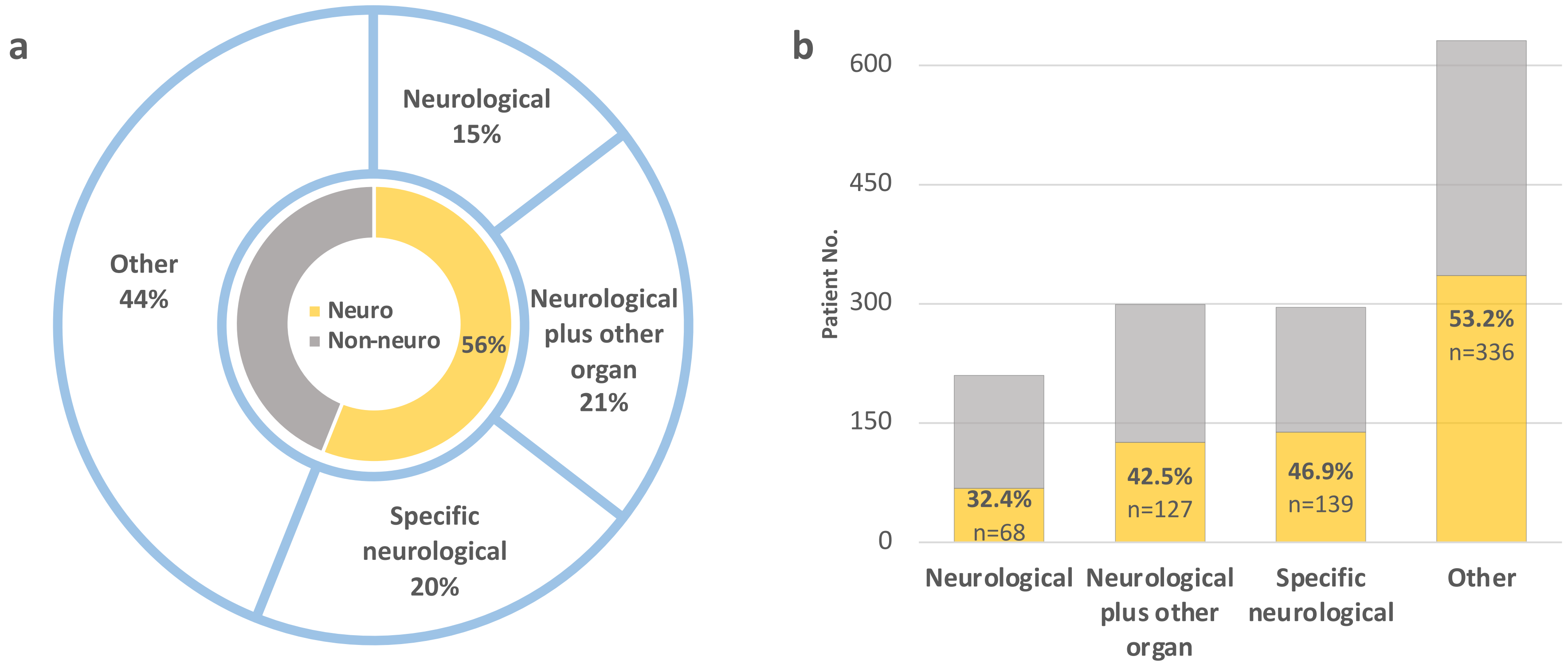

Supplementary Figure 2. Overview of phenotype distribution and diagnostic yield.

a Distribution of neurological phenotypes among 1436 patients. The neurological group included 210 patients with clinical presentations restricted to developmental delay, intellectual disability, seizures, and autism spectrum disorders. The neurological plus other organ systems group consisted of 299 patients presenting neurological findings along with at least one finding from a different organ system or multiple congenital anomalies. The specific neurological group included 296 patients with more defined neurological symptoms, such as spinal muscular atrophy, amyotrophic lateral sclerosis, hereditary spastic paraplegia, ataxia, parkinson's disease, sensorineural hearing loss, and peripheral neuropathy. The non-neurological group included 631 patients with a variety of conditions, including disorders of the muscle, eye, blood, connective tissue, skin, skeletal, and genitourinary systems. **b** Diagnostic rate in neurological and non-neurological patients.

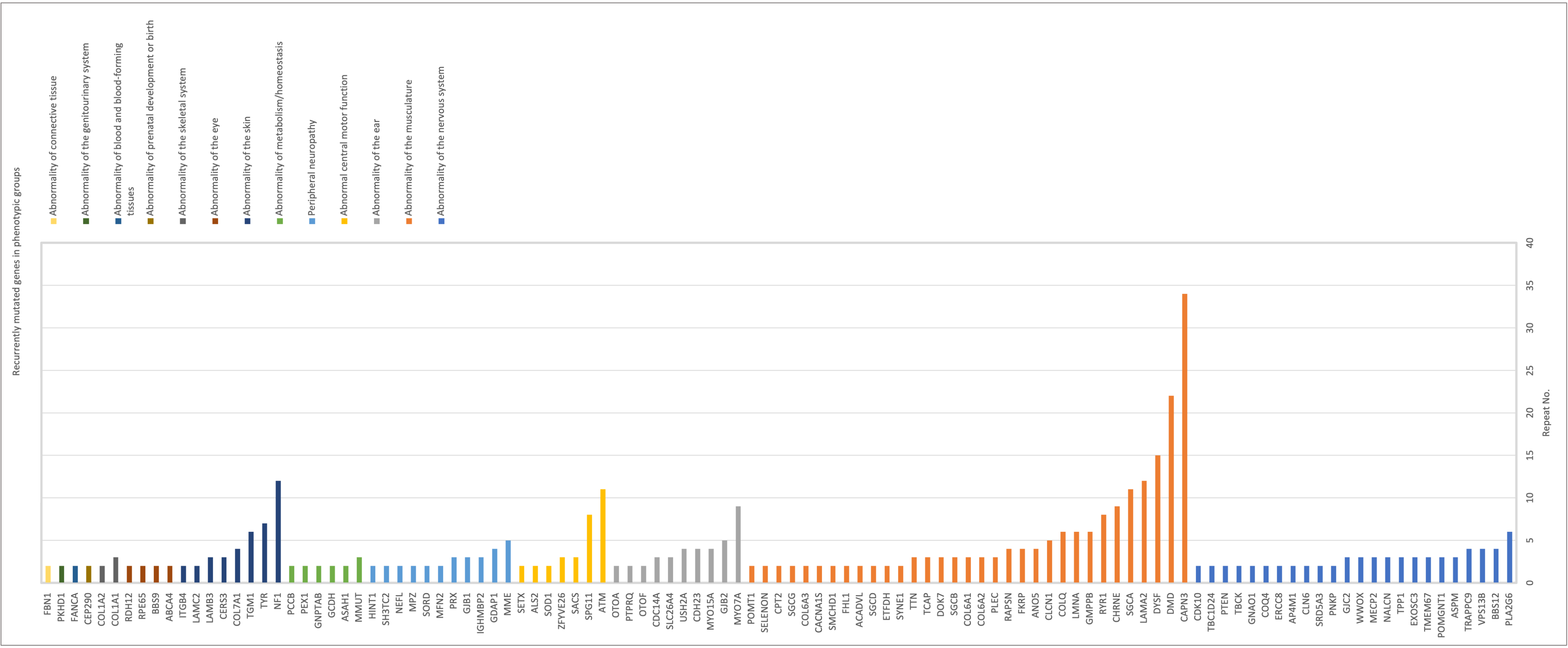

Supplementary Figure 3. Recurrently mutated genes in each phenotypic group.
